# Supplementary material for: Capsid integrity quantitative PCR to determine virus infectivity in environmental and food applications – A systematic review
Source: Water Res X. 2020 Dec 9;11:100080. doi: 10.1016/j.wroa.2020.100080 (PMC7811166; doi:10.1016/j.wroa.2020.100080)
Supplement: Multimedia component 1 [file mmc1.docx]

**SUPPLEMENTARY MATERIAL:**

**Capsid integrity quantitative PCR to determine virus infectivity in environmental and food applications – a systematic review**

Mats Leifels^a*^, Cheng Dan^a^, Emanuele Sozzi^b^, David C. Shoults^c^, Stefan Wuertz^a,d^, Skorn Mongkolsuk^e,f^, Kwanrawee Sirikanchana^e,f^

^a^ Singapore Centre for Environmental Life Sciences Engineering, Nanyang Technological University, Singapore (NTU), Singapore

^b^ Gilling’s School of Global Public Health, Department of Environmental Science and Engineering, University of North Carolina at Chapel Hill, NC, USA

^c^ Civil and Resource Engineering, Dalhousie University, Halifax, Nova Scotia, Canada

^d^ School of Civil and Environmental Engineering, NTU, Singapore

^e^ Research Laboratory of Biotechnology, Chulabhorn Research Institute, Bangkok, Thailand

^f^ Center of Excellence on Environmental Health and Toxicology, CHE, Ministry of Education, Bangkok, Thailand

*Corresponding author, email: [mats.leifels@rub.de](about:blank)

**Supplementary Table 1: Inclusion and Exclusion criteria and search strings for database search**

| **Inclusion Criteria** | **Search Strings** | **Exclusion Criteria** |
| --- | --- | --- |
| Viability RT-qPCR |  | bacteria |
| Viability PCR | ***PCR** | Archaea |
| Intercalating dyes |  | protozoa |
| *Virus | ***Virus** |  |
| *Phage | ***Phage** |  |
| Propidium Monoazide | ***azide** |  |
| Ethidium Monoazide |  |  |
| RT-qPCR |  |  |
| PMAx* | **PMA*** |  |
| **Infectivity** |  |  |
| PMA-RT-qPCR |  |  |
| EMA-RT-qPCR |  |  |
| EMA | **EMA** |  |
| PMA |  |  |
| **Polymerase Chain Reaction** |  |  |
| **capsid** |  |  |
| **viability** |  |  |

**Supplementary Table 2: Articles included in the Literature Review**

| **Publication** | **Virus** | | | **Reagent** | | **Incubation** | | **Light source** | | **Reference** |
| --- | --- | --- | --- | --- | --- | --- | --- | --- | --- | --- |
| Title | Type | Culture origin | Inactivation or treatment | Type | Concentration | Time | Temperature | Type | Duration |  |
| Discrimination of infectious bacteriophage T4 virus by Propidium Monoazide real-time PCR | Phage t4 | DSMZ culture | heat (85°C) and proteolysis | PMA in 20% DMSO | 100µM in 200µl | 5min | RT | LED (Led-Active Blue, system, Spain) | 15min | [1] |
| Application of viability PCR to discriminate the infectivity of hepatitis A virus in food samples, | Hepatitis A Virus (HM-175 Atcc VR-1402) | vegetable and shellfish concentrates | partially (99°C for 5min as negative control) | PMA and EMA | 50µM to 100µl concentrate | 10min | RT | LED (Led-Active Blue, Geniul) | 15min | [2] |
| Discrimination of Infectious Hepatitis A Viruses by Propidium Monoazide Real-Time RT-PCR, | Hepatitis A Virus (HM-175 Atcc VR-1402) | No information | heat inactivated | PMA in 20% DMSO | 50µM and 100µM final concentration | 5min | RT | LED (Led-Active Blue, Geniul) | 15min | [3] |
| Use of ethidium Monoazide and Propidium Monoazide to determine viral infectivity upon inactivation by heat, UV- exposure and chlorine, | HAdV5, Poliovirus 1 Sabin, Rotavirus WA, MNV, PhiX174 | culture | partially and completely by heat (45, 55, 65°C), UV and Chlorine (2mg/l) | PMA in 20% DMSO and EMA in 20% DMSO | 100µM in 200µl | 5min | RT | High Light (650W) on Ice | 5min | [4] |
| Using Propidium Monoazide to distinguish between viable and nonviable bacteria, MS2 and murine norovirus, | MS2 and MNV | culture | 10% Isopropanol of 10m, MNV 72°C for 10min, MS 80°C for 20min | PMA in 20%DMSO | 125µM or 250µM for MNC, 10µM, 50µM and 125µM for MS2 in 500µl | 5min | RT | Not defined  "Light exposure" | 10min | [5] |
| Ethidium Monoazide does not inhibit RT-PCR amplification of nonviable avian influenza RNA | Avian Influenza RNA | Cultured Virus mixed with water or landfill leachate | Decay Curve | EMA | 100µM | 5min | RT | High Light (500W) | 10min | [6] |
| Discrimination of infectious hepatitis A virus and rotavirus by combining dyes and surfactants with RT-qPCR | Hepatitis A virus (HM175/18f clone B) and Rotavirus SA11 | culture | Heat (37°C in water and 68°C, 72°C and 80°C in Dry heat) | PMA and EMA | 50µM in 100µl | 2h | 4°C | LED (LED-Active blue system) | 15min | [7] |
| Evaluation of viability PCR performance for assessing norovirus infectivity in fresh-cut vegetables and irrigation water | Norovirus G1P4 and GII.4 | stool samples in PBS + 2M NaNO3, 1% beef extract and 0.1% Triton | 99°C for 5min as negative controlee | EMA and PMA in 20% DMSO + PEMAX (Geniul Spain) and PMAxx (Biotinum) | 50µM for PMA, PEMAX and PMAxx and 20µM for EMA | 10min | RT | LED (LED Active blue) | 15min | [8] |
| Capsid-Damaging Effects of UV Irradiation as Measured by Quantitative PCR Coupled with Ethidium Monoazide Treatment | HAdV 5 and Poliovirus 1 | culture | UV | EMA in Milli-Q water | 50µM final | 30min | 4°C | High light (650W lamp) on ice | 3min | [9] |
| Viral persistence in surface and drinking water: Suitability of PCR pre-treatment with intercalating dyes (******) | AdV41 and Coxsackie B2 | surface water and decay curve | UV and Chlorine | EMA and PMA in molecular grade water | 2µM in 100µl | 30min | on ice | LED light (PhaST Blue System) | 15min | [10] |
| A very comprehensive and applied study on the decay of viruses in surface water in a temperate climate and the ability of capsid integrity qPCR to determine virus infectivity. The authors could show that pretreatment can help improve the assessment of water samples meant for consumption and that depending on the matrix and certain viruses, lower dye concentrations can result in excellent removal of false positives. | | | | | | | | | | |
| Detection of viable murine norovirus using the plaque assay and Propidium-Monoazide-combined real-time reverse transcription-polymerase chain reaction | MNV | culture | Heat inactivation (65 - 90°C for 1min) | EMA and PMA | 250µM PMA and 25µM EMA in 500µl | 10min | RT | LED light of 460nm wavelength | 15min at RT | [11] |
| Propidium Monoazide (PMA) and ethidium bromide Monoazide (EMA) improve DNA array and high-throughput sequencing of porcine reproductive and respiratory syndrome virus identification | PRRSV (porcine reproductive and respiratory syndrome virus) | lung and blood samples were spiked | no | EMA and PMA in RNAse free Water | 100µM final | 5min | RT | high light (500W light bulb) | 10min on ice | [12] |
| Propidium Monoazide Coupled with PCR Predicts Infectivity of Enteric Viruses in Swine Manure and Biofertilized Soil | HAdV2, rotavirus A, mengovirus and porcine Adenovirus | raw swine manure, swine affluent from anaerobic biodigester and Biofertilized soils | no | PMA | 50µM final concentration in 100µl | 10min | 25°C | LED (40W light at 460nm Wavelength) | 15min | [13] |
| Propidium Monoazide RT-qPCR assays for the assessment of hepatitis A inactivation and for a better estimation of the health risk of contaminated waters | Hepatitis A virus (pHM175 43c) | culture (originally from CDC) | Heat (70, 85, 99°C for 5min each) and Hypochlorite (0, 2,5, 5 and 10 mg/l for 30min) | PMA in water | 0 - 200µM with Triton if advised | 5min | RT (shaking) | LED (LED Active blue) | 15min | [14] |
| From Lab to Lake – Evaluation of Current Molecular Methods for the Detection of Infectious Enteric Viruses in Complex Water Matrices in an Urban Area | HAdV, Rotavirus and Enterovirus | Environment | no (but post UV sewage treatment was analyzed) | PMA and EMA in 20%DMSO | 40µM in 200µl Sample | 30min | on ice | LED (Led-Active Blue, Geniul) | 15min | [15] |
| Use of Propidium Monoazide in Reverse Transcriptase PCR To Distinguish between Infectious and Noninfectious Enteric Viruses in Water Samples▿ | Poliovirus type 1, echovirus 7, coxsackie-virus b2 and Norwalk Virus | culture and stool for Norwalk, also concentrated environmental water as a matrix | heat inactivation (19°C, 37°C and 72°C for 5 - 6min) and chlorine (0.5mg/l) | PMA in 20%DMSO | 25µM in 100µl of sample | 5min | unknown | high light (650 W light) | 3min | [16] |
| Thermal inactivation of human norovirus on spinach using Propidium or ethidium Monoazide combined with real-time quantitative reverse transcription-polymerase chain reaction | Norovirus GII.4 | originated from stool samples and was added to spinach | heat inactivation between 65°C - 85°C for 1min | PMA and EMA | 250µM PMA and 25µM EMA in 500µl | 10min | RT | LED (40W light at 460nm Wavelength) | 15min at RT | [17] |
| Evaluation of Propidium Monoazide and long-amplicon qPCR as an infectivity assay for coliphage | MS2 phages | culture | chlorination and UV treatment | PMA | 10µM | 5min | RT (shaking) | high light (500W light bulb) | 10min | [18] |
| Enzymatic and viability RT-qPCR assays for evaluation of enterovirus, hepatitis A virus and norovirus inactivation: Implications for public health risk assessment | Norovirus GII.4, Hepatitis A virus HM175/18f, Coxsackie B3 | culture and stool for Norovirus | Heat (95°C for 10min) and enzymatic digestion | Reagent D (Biotecon Diagnostics Germany) in 1:4 proportion | unknown | 5min | RT | LED (PhaST Blue LED System) | 15min | [19] |
| Evaluation of Assays to Quantify Infectious Human Norovirus for Heat and High-Pressure Inactivation Studies Using Tulane Virus | Tulane Virus and Norovirus GII.4 and GI.1 | No info | Heat (60 - 90°C for 2min) and High Pressure | PMA in ddH2O | 100µM | 15min | RT | LED (Biotium PMA-Lite Device) | 10min | [20] |
| Molecular methods used to estimate thermal inactivation of a prototype human norovirus: More heat resistant than previously believed | Norovirus (Snow Mountain Virus GII.2) | stool sample | heat (72 - 90°) | PMA | 100µM | 60min | RT | high light (500W light bulb) | 5min | [21] |
| Propidium Monoazide reverse transcriptase PCR and RT-qPCR for detecting infectious enterovirus and norovirus | Norwalk Virus, Poliovirus 1, MNV1 | stool sample and culture | heat (72°C - 99°C) and chlorine (0.5mg/l for 0.5 - 20min) and UV | PMA in 20% DMSO | 25µM in 100µl Sample | 5min | RT | High light (800W lamp) | 3min | [22] |
| Optimization of PMAxx pretreatment to distinguish between human norovirus with intact and altered capsids in shellfish and sewage samples | Norovirus GI-P4 and Noro GI.1 as well as Noro GII.4 and Noro GII.3 from fecal samples and Mengovirus MC0 as a control | stool samples (Noro) and Culture (Mengo) | heat inactivation (60°C, 72°C and 95°C for 15 Min) in bio accumulated oysters, sewage and PBS | PMAxx | 50µM PMAxx in 0.5% Triton for PBS control and sewage and 100µM PMAxx in 0.5% Triton for oysters | 10 Min | RT (shaking) | LED light (464 - 476nm) | 15min | [23] |
| Improving efficiency of viability‐qPCR for selective detection of infectious HAV in food and water samples | Hepatitis Virus A (HM-175 18f) and Mengovirus in Sewage samples | culture (HeLa and FRhK-4 Cells) | heat inactivation (60°C, 72°C and 95°C for 15 Min) | PMA, PMAxx and PEMAX | 50µM of all dyes | 10 Min | RT (shaking) | LED light system (464 - 476nm; Geniul) | 15 Min | [24] |
| Pretreatment with Propidium Monoazide/sodium lauroyl sarcosinate improves discrimination of infectious waterborne virus by RT-qPCR combined with magnetic separation | Norovirus GII.4, PMMoV | patient stool (Noro), not clear about PMMoV but most likely culture | Noro inactivated with 1 - 50mg/l chlorine | PMA with 0.1 - 1.0% lauroyl sarcosinate (sigma) as a detergent | 10, 50, 100, 200 and 300µM of PMA | 5 min | RT (in the dark) | LED (45W; 464nm; PhaST Blue System) | 15 min | [25] |
| Impact of Various Humic Acids on EMA-RT-qPCR to Selectively Detect Intact Viruses in Drinking Water | Aichi Virus (both intact virions and naked nucleic acids) | Cell culture (BGM cells) | no inactivation but addition of four commercially available Humic Acids in powder form | EMA in ddH20 | 50µg/ml | 30 min | 4°C in the dark | 650-Watt halogen lamp at 15cm and on ice | 3min | [26] |
| Performance of pre-RT-qPCR treatments to discriminate infectious human rotaviruses and noroviruses from heat-inactivated viruses: applications of PMA/PMAxx, benzonase and RNAse (*****) | Rotavirus Wa G1P and Noro-/Rotavirus from Patient stool | cell culture (G1P; MA104) and patient stool | 80°C for 1 - 30 min a water bath | PMA, PMAxx, Benzonase and RNAse | 100µmol l-1 for PMA and PMAxx, 100U of benzonase per 100µl aliquot and 80U of Ribolock RNAse | 60 min | 6°C in the dark | LED (Biotium-Lite LED Photolysis device) | 15 min at RT | [27] |
| The researchers could show that all pretreatments resulted in promising trends to reduce presumably false positive signals from in both cell culture medium and diluted stool samples. | | | | | | | | | | |
| Discrimination of infectious and heat-treated norovirus by combining platinum compounds and real-time RT-PCR | Human Norovirus, murine norovirus | Noro (GI.2,3,4,6 and GII.3,4,17) from stool samples, MNV-1 propagated on RAW cells | 56°C, 72°C and 80°C for a not defined time | EMA/PMA/PMAxx in RNAse free water and Platinum/Palladium Compounds in 20%DMSO | 20, 50, 100, 250µM PMA and 50/1,000/2,500µM Platinum Compounds (no info on Palladium compounds) | 30 min | 5°C in the dark | LED-Active Blue System | 15min | [28] |
| Detection of Infectious Noroviruses from Wastewater and Seawater Using PEMAX TM Treatment Combined with RT-qPCR (******) | NoroV GI.3 / GII.4 and MNV | MNV cultured on RAW Cells, NoroGI.3/GII.4 from patient stool | 90°C for 3 min | PMAX in 20% DMSO | concentrations of 50µM, 100µM or 200µM | 30 min | no temperature given, in the dark | LED (PhaST Blue LED System) | 15min | [29] |
| The authors of this study could show that heat-inactivated NoroG1.3 and NoroGII.4 could be removed better using PMAxx than conventional qPCR (0.34 - 0.98 log for GI.3, 0.63 - 2.06 log for GII.4). Has been successfully adopted for highly complex environmental sample matrices like sewage and seawater. | | | | | | | | | | |
| Irrigating Lettuce with Wastewater Effluent: Does Disinfection with Chlorine Dioxide Inactivate Viruses | Norovirus GI.P4/GII.4 and Astrovirus | present in the reclaimed wastewater used for irrigation of green leaf salad | chlorination of the greywater (approx. 6,000 mg/l-1) | PMAxx in 0.5% Triton | 50µM in 100µl | 10 min | dark room at room temperature and in a shaker at 150 rpm | LED Active Blue (PhaST blue) | 15 min | [30] |
| Viability RT-qPCR Combined with Sodium Deoxycholate Pre-treatment for Selective Quantification of Infectious Viruses in Drinking Water Samples | Aichivirus 1 as a representative of human enteric virus | not listed, most likely culture | heat inactivation at 50, 60, 70, 80 and 90°C for 1min and initial concentration of 1mg/l Chlorine for 1, 2, 5 or 10min | PMA, EMA and CDDP | 100µM and 1,000µM | 30 Min | 4°C in the dark | 650-Watt lamp at distance of 15cm | 3min | [31] |
| Simultaneous Detection of Selected Enteric Viruses in Water Samples by Multiplex Quantitative PCR | Rotavirus, HAdV 40/41 and Norovirus | Rotavirus WA (ATCC VR-2018), HAdV 40/41 (Dugan / Tak Strains ATCC) and Norovirus (no G mentioned) isolated from clinical samples | no treatment | PMA | 10 µM final concentration | 10 Min | 20-26°C in the dark | halogen lamp (most likely 500 - 650 W) | 10 min on Ice | [32] |
| Detection of infectious dengue virus by selective real-time quantitative polymerase chain reaction (*****) | Dengue Virus Serotype 2 | patients infected with that virus | Temperature between 56 -100°C) for 30 minutes | PMA | 50 µM per liter in Water | 5 min | 20°C in the dark with occasional mixing | LED Lamp emitting blue light | 15 min on ice | [33] |
| This nuance study was the first to utilize dengue virus originating from infected patients for capsid integrity qPCR. Treatment with PMA showed similar results to non-PMA qPCR if used for patients infected with the virus in acute phase (virus likely to be capsid intact and infectious) and a great ability to remove non-infectious (false-positive) virions after heat inactivation of 70°C or more. | | | | | | | | | | |
| Use of RT-qPCR with combined intercalating dye and sodium lauroyl sarcosinate pretreatment to evaluate the virucidal activity of halophyte extracts against norovirus | Human Norovirus GII.4 | Stool samples suspended in RNAse-free water | inactivation with five plant compounds (50 and 100 µg/ml) and hypochlorite as positive control | PMA with 0.1 - 1.0% lauroyl sarcosinate (sigma) as detergent | 10, 50, 100, 200 and 300µM of PMA | 5 min | RT (in the dark) | LED (45W; 464nm; PhaST Blue System) | 15 min | [34] |
| Propidium Monoazide Integrated with qPCR Enables the Detection and Enumeration of Infectious Enteric RNA and DNA Viruses in Clam and Fermented Sausages | Mengovirus and HAdV 2 | origin not mentioned but cultured on A549 and HeLa | 10 min at 95°C as a control for the food item spiking | PMA | 50µM final concentration | 10 min | 25°C in dark room | 40 W LED light emitting light at 460nm (PhastBlue) | 15 min at RT | [35] |
| A combined treatment of UV-assisted TiO2 photocatalysis and high hydrostatic pressure to inactivate internalized murine norovirus | murine Norovirus CW 1 | culture | UV assisted TiO2 (0 -20min) and high hydrostatic pressure (300 - 600 Mpa for 1 - 15min at RT) both combined and separately | PMA | 200µM in 100µl of sample | 60 min | dark room with shaking at 25°C | 60 W LED light | 30min | [36] |
| Viability RT-qPCR to Distinguish Between HEV and HAV With Intact and Altered Capsids | Hepatitis Virus E genotype 3f and HAV HM-175/18f | HEV provided by hospital, most likely patient and HAV from ATCC | 60, 72 and 95°C for 15min and High-Pressure Processing (600 Mpa) | PMAxx and PtCl4 | 50, 100 and 250 µM | 10 Min | shaker at RT | LED Active Blue System | 15min | [37] |
| Interlaboratory Comparative Study to Detect Potentially Infectious Human Enteric Viruses in Influent and Effluent Waters (******) | fecal Norovirus GI / GII, fecal Astrovirus, Hepatitis A Virus, HM-175, Rotavirus Wa and Mengovirus | fecal (NoV GI/GII, HAstV) and culture | environmental samples, no inactivation | PMAxx | 50µM final concentration with 7.7mmol/L triton100-x | 10 Min | room temperature in a shaker at 150rpm | LED Active Blue System | 15min | [38] |
| PMAxx treatment of environmental water samples succeeded in significantly decrease virus titers but comparably high concentrations remained, indicating infectious virions in both influent and effluent. Result indicate well know bias towards UV disinfected waters and inability of PMA based reagents to pick up this kind of inactivation. Very good study design to evaluate the effect of conducting analysis in different laboratories. | | | | | | | | | | |
| Capsid integrity qPCR – an azo-dye based and culture-independent approach to estimate adenovirus infectivity after disinfection and in the aquatic environment | HAdV 5. murine Norovirus | Culture | UV-C inactivation 150 mJxcm2, 95°C for 10min, 2mg/L hypochlorite | PMA or EMA | 10µM in 200µl of diluted Sample | 30 Min | in the Dark on ice | LED Active Blue System | 15min | [39] |
| Evaluating Microbial and Chemical Hazards in Commercial Struvite Recovered from Wastewater | Norovirus GII, human Adenovirus and Entero-viruses | Environment Samples (Struvite; reclaimed phosphate from sewage) | Inactivation during Struvite production (presumably electro-chemical precipitation) | PMA | 10µM in 200µl of diluted sample | 30 Min in the dark followed by 15min daylight for photo induced cross linking | Room temperature | 4-W LED lamp | 15min | [40] |
| Viability RT-qPCR to detect potentially infectious enteric viruses on heat-processed berries | NoV GI / GII, HAV HM175/18f and Mengovirus | Environ-mental Noro, cultured HAV and Mengo | untreated (room temperature) as well as 15min at 60°C, 72°C and 95°C | PMAxx and Triton X-100 | 50 and 100µM | 10 min or 30 min | 4°C and room temp on shaker | LED Active Blue System | 15min or 30min | [41] |

**References:**

1. Fittipaldi, M., A. Nocker, and F. Codony, *Progress in understanding preferential detection of live cells using viability dyes in combination with DNA amplification.* Journal of Microbiological Methods, 2012. **91**(2): p. 276-289.

2. Moreno, L., R. Aznar, and G. Sanchez, *Application of viability PCR to discriminate the infectivity of hepatitis A virus in food samples.* Int J Food Microbiol, 2015. **201**: p. 1-6.

3. Sánchez, G., P. Elizaquível, and R. Aznar, *Discrimination of Infectious Hepatitis A Viruses by Propidium Monoazide Real-Time RT-PCR.* Food and Environmental Virology, 2012. **4**(1): p. 21-25.

4. Leifels, M., et al., *Use of ethidium monoazide and propidium monoazide to determine viral infectivity upon inactivation by heat, UV- exposure and chlorine.* International Journal of Hygiene and Environmental Health, 2015. **218**(8): p. 686-693.

5. Kim, S.Y. and G. Ko, *Using propidium monoazide to distinguish between viable and nonviable bacteria, MS2 and murine norovirus.* Lett Appl Microbiol, 2012. **55**(3): p. 182-8.

6. Graiver, D.A., et al., *Ethidium monoazide does not inhibit RT-PCR amplification of nonviable avian influenza RNA.* J Virol Methods, 2010. **164**(1-2): p. 51-4.

7. Coudray-Meunier, C., et al., *Discrimination of infectious hepatitis A virus and rotavirus by combining dyes and surfactants with RT-qPCR.* BMC Microbiology, 2013. **13**(1): p. 216.

8. Randazzo, W., et al., *Evaluation of viability PCR performance for assessing norovirus infectivity in fresh-cut vegetables and irrigation water.* Int J Food Microbiol, 2016. **229**: p. 1-6.

9. Sangsanont, J., et al., *Capsid-Damaging Effects of UV Irradiation as Measured by Quantitative PCR Coupled with Ethidium Monoazide Treatment.* Food and Environmental Virology, 2014. **6**(4): p. 269-275.

10. Prevost, B., et al., *Viral persistence in surface and drinking water: Suitability of PCR pre-treatment with intercalating dyes.* Water Research, 2016. **91**: p. 68-76.

11. Lee, M., et al., *Detection of viable murine norovirus using the plaque assay and propidium-monoazide-combined real-time reverse transcription-polymerase chain reaction.* J Virol Methods, 2015. **221**: p. 57-61.

12. Bellehumeur, C., et al., *Propidium monoazide (PMA) and ethidium bromide monoazide (EMA) improve DNA array and high-throughput sequencing of porcine reproductive and respiratory syndrome virus identification.* J Virol Methods, 2015. **222**: p. 182-91.

13. Fongaro, G., et al., *Propidium Monoazide Coupled with PCR Predicts Infectivity of Enteric Viruses in Swine Manure and Biofertilized Soil.* Food Environ Virol, 2016. **8**(1): p. 79-85.

14. Fuster, N., et al., *Propidium monoazide RTqPCR assays for the assessment of hepatitis A inactivation and for a better estimation of the health risk of contaminated waters.* Water Research, 2016. **101**: p. 226-232.

15. Leifels, M., et al., *From Lab to Lake – Evaluation of Current Molecular Methods for the Detection of Infectious Enteric Viruses in Complex Water Matrices in an Urban Area.* PLOS ONE, 2016. **11**(11): p. e0167105.

16. Parshionikar, S., I. Laseke, and G.S. Fout, *Use of propidium monoazide in reverse transcriptase PCR to distinguish between infectious and noninfectious enteric viruses in water samples.* Applied and environmental microbiology, 2010. **76**(13): p. 4318-4326.

17. Jeong, M.-I., S.Y. Park, and S.-D. Ha, *Thermal inactivation of human norovirus on spinach using propidium or ethidium monoazide combined with real-time quantitative reverse transcription-polymerase chain reaction.* Food Control, 2017. **78**: p. 79-84.

18. McLellan, N.L., H. Lee, and M.B. Habash, *Evaluation of propidium monoazide and long-amplicon qPCR as an infectivity assay for coliphage.* J Virol Methods, 2016. **238**: p. 48-55.

19. Monteiro, S. and R. Santos, *Enzymatic and viability RT-qPCR assays for evaluation of enterovirus, hepatitis A virus and norovirus inactivation: Implications for public health risk assessment.* J Appl Microbiol, 2018. **124**(4): p. 965-976.

20. Li, X., R. Huang, and H. Chen, *Evaluation of Assays to Quantify Infectious Human Norovirus for Heat and High-Pressure Inactivation Studies Using Tulane Virus.* Food Environ Virol, 2017. **9**(3): p. 314-325.

21. Escudero-Abarca, B.I., et al., *Molecular methods used to estimate thermal inactivation of a prototype human norovirus: more heat resistant than previously believed?* Food Microbiol, 2014. **41**: p. 91-5.

22. Karim, M.R., et al., *Propidium monoazide reverse transcriptase PCR and RT-qPCR for detecting infectious enterovirus and norovirus.* Journal of Virological Methods, 2015. **219**: p. 51-61.

23. Randazzo, W., et al., *Optimization of PMAxx pretreatment to distinguish between human norovirus with intact and altered capsids in shellfish and sewage samples.* Int J Food Microbiol, 2018. **266**: p. 1-7.

24. Randazzo, W., et al., *Improving efficiency of viability-qPCR for selective detection of infectious HAV in food and water samples.* J Appl Microbiol, 2018. **124**(4): p. 958-964.

25. Lee, H.W., et al., *Pretreatment with propidium monoazide/sodium lauroyl sarcosinate improves discrimination of infectious waterborne virus by RT-qPCR combined with magnetic separation.* Environ Pollut, 2018. **233**: p. 306-314.

26. Canh, V.D., et al., *Impact of Various Humic Acids on EMA-RT-qPCR to Selectively Detect Intact Viruses in Drinking Water.* Journal of Water and Environment Technology, 2018. **16**(2): p. 83-93.

27. Oristo, S., H.-J. Lee, and L. Maunula, *Performance of pre-RT-qPCR treatments to discriminate infectious human rotaviruses and noroviruses from heat-inactivated viruses: applications of PMA/PMAxx, benzonase and RNase.* Journal of Applied Microbiology, 2018. **124**(4): p. 1008-1016.

28. Fraisse, A., et al., *Discrimination of infectious and heat-treated norovirus by combining platinum compounds and real-time RT-PCR.* Int J Food Microbiol, 2018. **269**: p. 64-74.

29. Gyawali, P. and J. Hewitt, *Detection of Infectious Noroviruses from Wastewater and Seawater Using PEMAXTM Treatment Combined with RT-qPCR.* Water, 2018. **10**(7): p. 841.

30. Lopez-Galvez, F., et al., *Irrigating Lettuce with Wastewater Effluent: Does Disinfection with Chlorine Dioxide Inactivate Viruses?* J Environ Qual, 2018. **47**(5): p. 1139-1145.

31. Canh, V.D., et al., *Viability RT-qPCR Combined with Sodium Deoxycholate Pre-treatment for Selective Quantification of Infectious Viruses in Drinking Water Samples.* Food Environ Virol, 2019. **11**(1): p. 40-51.

32. Lee, D.-Y., et al., *Simultaneous Detection of Selected Enteric Viruses in Water Samples by Multiplex Quantitative PCR.* Water, Air, & Soil Pollution, 2016. **227**(4): p. 107.

33. Huang, X., et al., *Detection of infectious dengue virus by selective real-time quantitative polymerase chain reaction.* Virologica Sinica, 2016. **31**(4): p. 342-345.

34. Lee, H.-W., et al., *Use of RT-qPCR with combined intercalating dye and sodium lauroyl sarcosinate pretreatment to evaluate the virucidal activity of halophyte extracts against norovirus.* Food Control, 2019. **98**: p. 100-106.

35. Quijada, N.M., et al., *Propidium Monoazide Integrated with qPCR Enables the Detection and Enumeration of Infectious Enteric RNA and DNA Viruses in Clam and Fermented Sausages.* Frontiers in microbiology, 2016. **7**: p. 2008-2008.

36. Kim, S.-H., et al., *A combined treatment of UV-assisted TiO2 photocatalysis and high hydrostatic pressure to inactivate internalized murine norovirus.* Innovative Food Science & Emerging Technologies, 2017. **39**: p. 188-196.

37. Randazzo, W., et al., *Viability RT-qPCR to Distinguish Between HEV and HAV With Intact and Altered Capsids.* Frontiers in microbiology, 2018. **9**: p. 1973-1973.

38. Randazzo, W., et al., *Interlaboratory Comparative Study to Detect Potentially Infectious Human Enteric Viruses in Influent and Effluent Waters.* Food and Environmental Virology, 2019. **11**(4): p. 350-363.

39. Leifels, M., et al., *Capsid Integrity qPCR—An Azo-Dye Based and Culture-Independent Approach to Estimate Adenovirus Infectivity after Disinfection and in the Aquatic Environment.* Water, 2019. **11**(6): p. 1196.

40. Yee, R.A., et al., *Evaluating Microbial and Chemical Hazards in Commercial Struvite Recovered from Wastewater.* Environmental Science & Technology, 2019. **53**(9): p. 5378-5386.

41. Chen, J., et al., *Viability RT-qPCR to detect potentially infectious enteric viruses on heat-processed berries.* Food Control, 2020. **107**: p. 106818.
